# Snow algae blooms are beneficial for microinvertebrates assemblages (Tardigrada and Rotifera) on seasonal snow patches in Japan

**DOI:** 10.1038/s41598-021-85462-5

**Published:** 2021-03-16

**Authors:** Masato Ono, Nozomu Takeuchi, Krzysztof Zawierucha

**Affiliations:** 1 Graduate School of Science and Engineering, Chiba University, Chiba, Japan; 2 Department of Earth Science, Graduate School of Science, Chiba University, Chiba, Japan; 3 Department of Animal Taxonomy and Ecology, Adam Mickiewicz University, Poznan, Poland

**Keywords:** Ecosystem ecology, Forest ecology, Freshwater ecology, Microbial ecology

## Abstract

Although studies on snow algae and macroinvertebrates have been frequently conducted on snow patches, only few surveys have been focused on microinvertebrates which reach high biomass and play various trophic roles in other cold habitats. The aims of this study were (1) to search for microinvertebrates in seasonal surface snow patches located on the slope of Mt. Gassan, in northern Japan, and (2) to identify factors determining their distribution associated with snow algal blooms of various colorations (orange, green, and golden-brown) collected from the same sampling site over two seasons (2018, 2019). Microscopic observation revealed presence of two major groups of microinvertebrates: Tardigrada and Rotifera. They were concentrated in green snow colored by blooms of *Chloromonas* sp. in comparison to orange or golden-brown snow and only a few were found in white snow. Mean body length of tardigrades increased throughout the melt season, their intestine content was green and they laid eggs on colored snow. These results suggest that tardigrades preferentially grew and reproduced on green snow patches. Population densities of tardigrades, rotifers and concentration of chlorophyll *a* were significantly correlated. Our study indicates that green snow patches in temperate mountainous forests constitute important and unique low-temperature ecosystems for microinvertebrates. Snow covered by algae is an unrecognized novel habitats for tardigrades and rotifers.

## Introduction

Seasonal snow is one of the most common, yet, at the same time, ephemeral cold environment in the world^1^. Because of its accumulation during winter and melting during the spring, seasonal snow influences adjacent ecosystems such as terrestrial, freshwater and marine ecosystems serving as a source of water or nutrients^2^. In some cases snow significantly affects the whole ecosystems. For example a reduction in seasonal snow might lead to soil freezing and subsequent damage to forest ecosystems^3^, or melting snow might play an important role in supporting nutrient input to glacial ecosystem and biological production there^4^.

Although snow patches are an important element supporting the functioning of other ecosystems, seasonal snow itself provides specific environments for microorganisms. Despite the harsh conditions of snow surface habitat such as a low temperature and high UV irradiation, there are many organisms inhabiting these unique ecosystems including primary producers (snow algae and cyanobacteria), microbial heterotrophs like fungi^5^, and consumers represented mostly by insects^6^. Activity of microorganisms in seasonal snow could influence its melting by reducing albedo^7^. As snow ecosystems are shrinking and disappearing globally, the efforts are needed to fully understand their productivity and biodiversity.

During the melt season, snow algae blooms change the color of snow surfaces into red, green, golden-brown or orange^8^. One of the most common genera of snow algae are *Chloromonas* and *Sanguina*, reported in glacial and snow habitat worldwide including Japan. *Sanguina* ssp. is common on open snowfields while *Chloromonas* ssp. often appears on more shaded forest floor^9–12^. Snow algae blooms have been shown to increase melt rates due to reduction of albedo of the snow surface, in consequence providing liquid water for biological processes^13,14^. Blooms are also an important component of nutrient and carbon cycles^7,15^. Potential consumers of algae (e.g. invertebrates) and heterotrophs (e.g. ciliates) also have been reported on snow patches^5,6,16,17^, but detailed ecological data explaining links between their densities, biomass, sources and seasonality are still missing. Although invertebrates are common apex consumers in snow ecosystems, their diversity was mostly reported for macroinvertebrates in high mountains^18,19^. Data on microinvertebrates, particularly tardigrades and rotifers which are one of the most common faunal groups in polar and high mountain ecosystems^20^, are still very limited.

Tardigrada and Rotifera are cosmopolitan, microscopic (mostly < 1 mm) invertebrates inhabiting almost all of terrestrial and aquatic ecosystems, from tropical rain forests to glaciers in polar regions^20,21^. Until now, ca.1400 and 2000 species of tardigrades and rotifers respectively have been described worldwide^22,23^. Their feeding mode and behaviors (herbivorous, omnivorous or carnivorous; grazers, filter-feeders, predators) and reproduction style (sexual, asexual and parthenogenetic) differ depending on the species^24^. They inhabit a wide range of environments worldwide, including hostile habitats like cryoconite holes on glaciers^25^, thanks to their ability of cryptobiosis which enables them to survive in extreme conditions such as high UV radiation, low and high temperature or salinity^26,27^.

Although there are many studies devoted to ecology and diversity of tardigrades and rotifers in high mountains or polar bryophytes and lichen habitats^28,29^, their cryospheric ecology is still underscored^30,31^. Even though snow ecosystems are common on both hemispheres, data on tardigrades and rotifers on snow are still missing^5^. Both groups play important role as consumers (being grazers and predators), not only in cryosphere but also in other ecosystems^32–35^. For example grazing may influence the life cycle and distribution of primary producers^33^. Limited data on microinvertebrates on snow may hinder our understanding of trophic networks and functioning of low temperature habitats. This study aimed to (1) determine the abundance and diversity of microinvertebrates, (2) identify the factors determining their distribution in the seasonal snow habitat under the tree canopy. Microinvertebrates were collected in the late melt season (2018 and 2019) in the Northern Japanese mountainous area. Population density, body size, biomass, diet and habitat preferences were measured. Additionally, local tree mosses were collected to evaluate whether tardigrade species inhabiting snow and mosses were similar.

For our survey, we chose Northern Japanese mountains due to the long history of research on snow ecosystems^9^. Thanks to influence of monsoon, a large amount of snow accumulates during the winter in mountainous areas in Japan. This accumulated snow remains in place until late spring and plays a role as a low temperature habitats for temperate psychrophiles that needed to spend a part or whole of their life in low temperature environments (e.g. stonefly^36^).

## Results

### Microinvertebrates on snow surface

**Figure 1:**
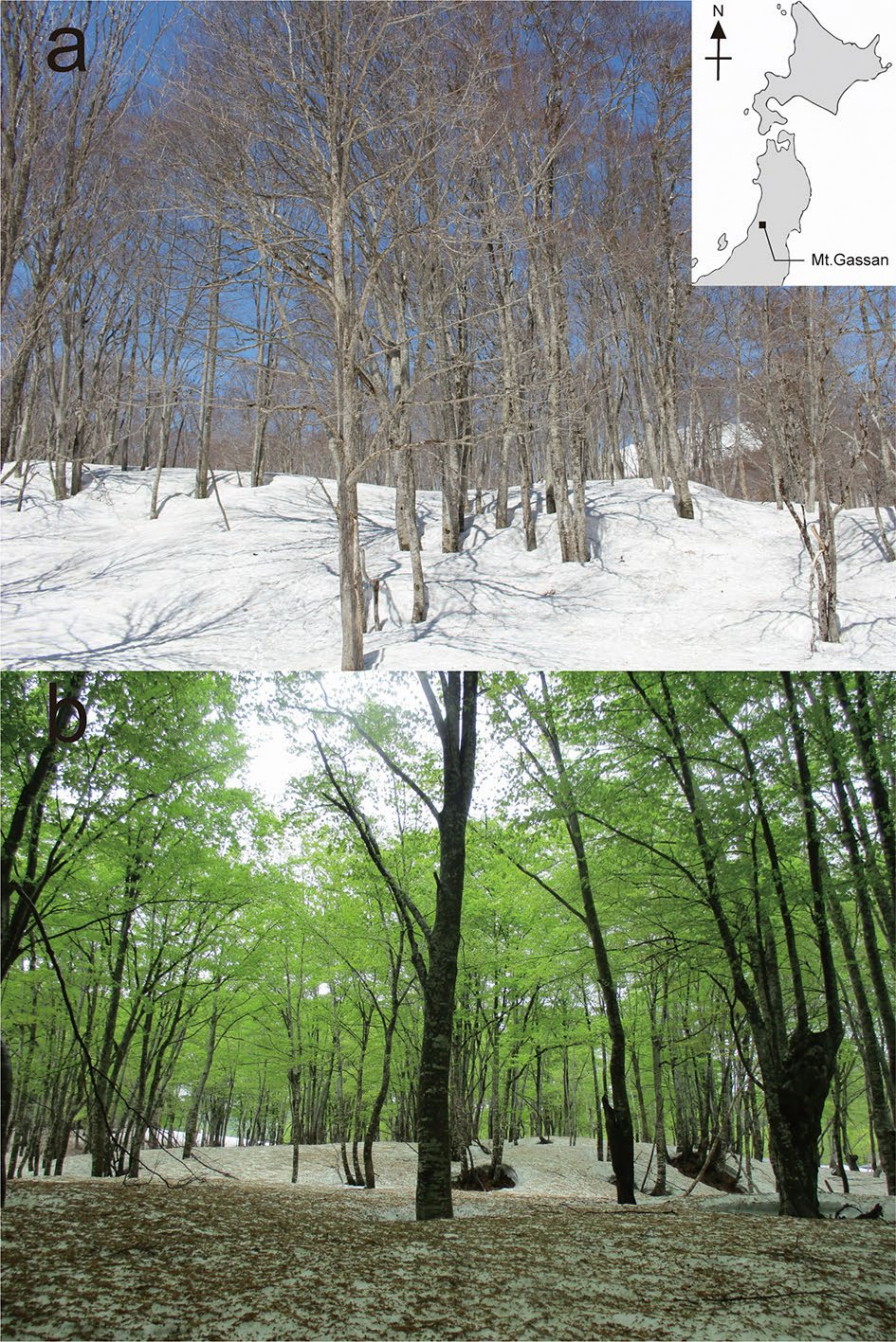
Study site (**a**) in April, 2018 when beech leaf trees did not leaf out yet, (**b**) in May, 2018 (same in May, 2019) when beech came into leaf opened and bud scales were present on snow (insert indicate study area in Japan).

**Figure 2:**
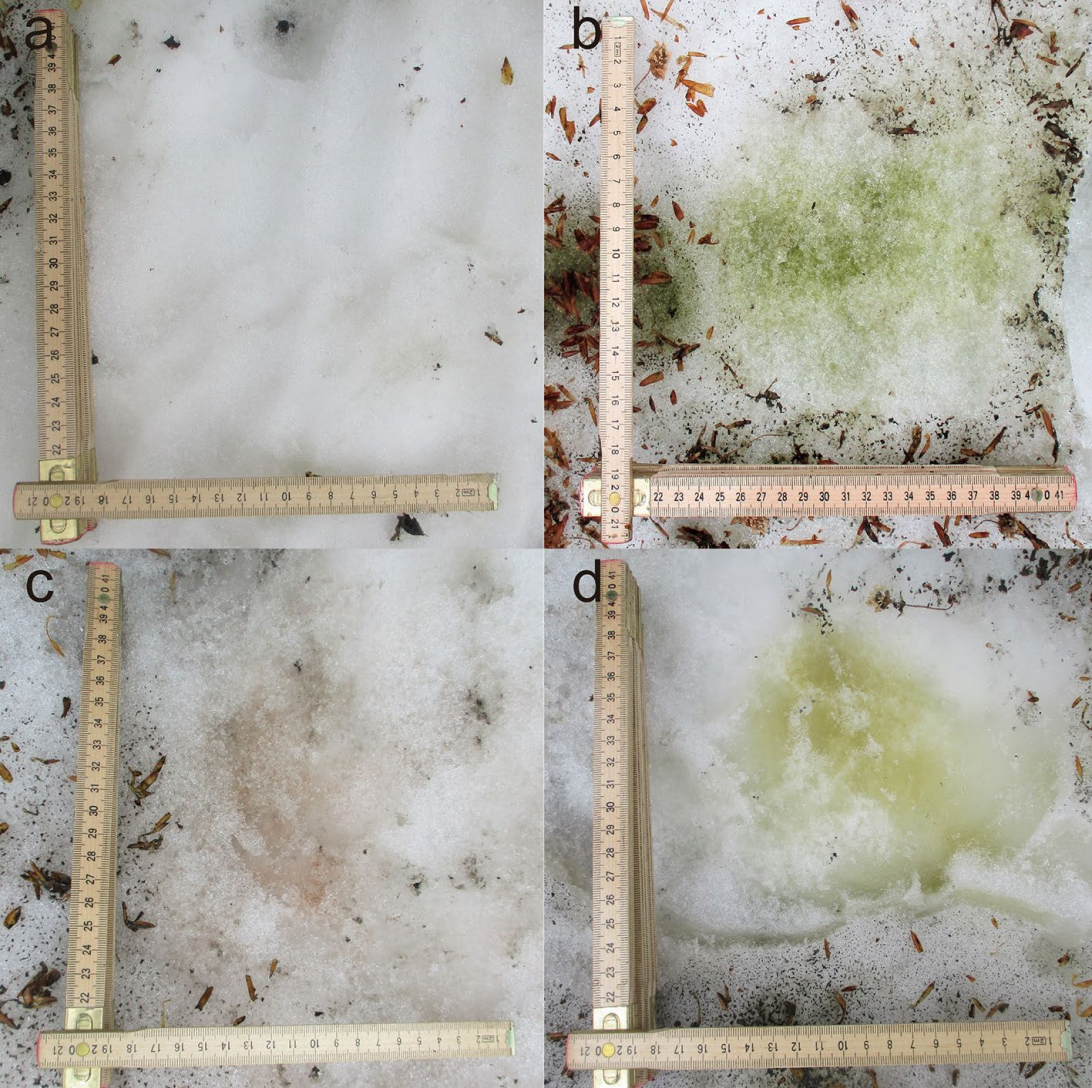
Snow surface collected in 2018. (**a**) White snow, (**b**) green snow, (**c**) orange snow, (**d**) golden-brown snow.

**Figure 3:**
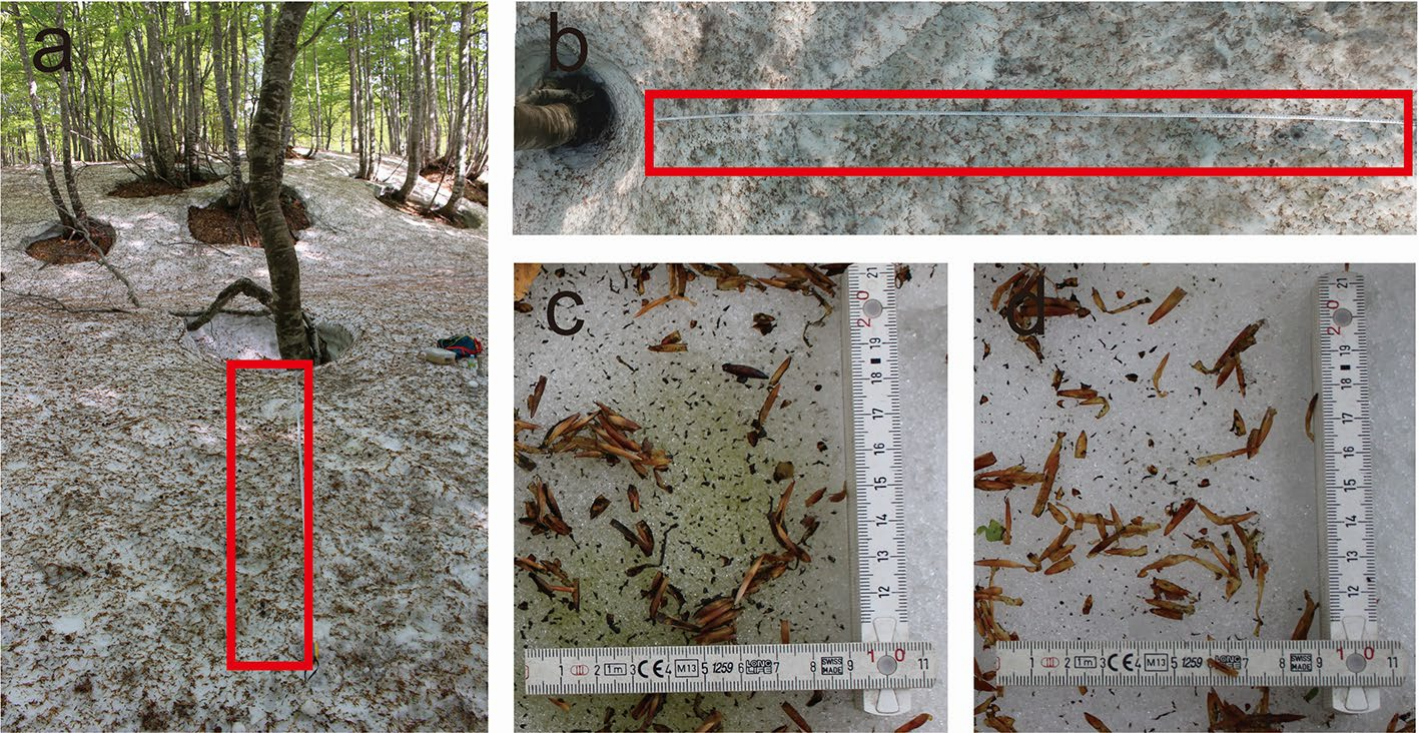
Snow surface collected in 2019. (**a**,**b**) Collection area along a lateral line (surrounded by red frame), (**c**) green snow, (**d**) white snow.

**Figure 4:**
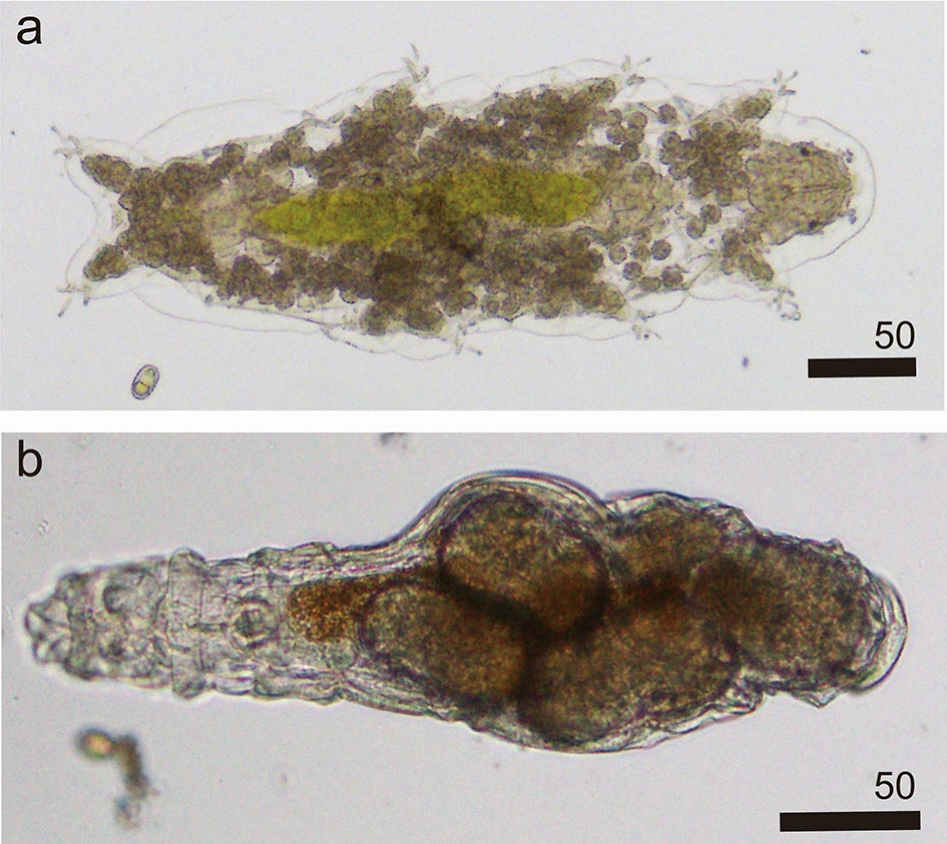
Microinvertebrates present in green snow collected in 2019. (**a**) *Hypsibius* sp. full of green pigment (digested algae) in their intestine (LM), (**b**) *Philodina* sp. (LM). All scale bars in micrometers.

**Figure 5:**
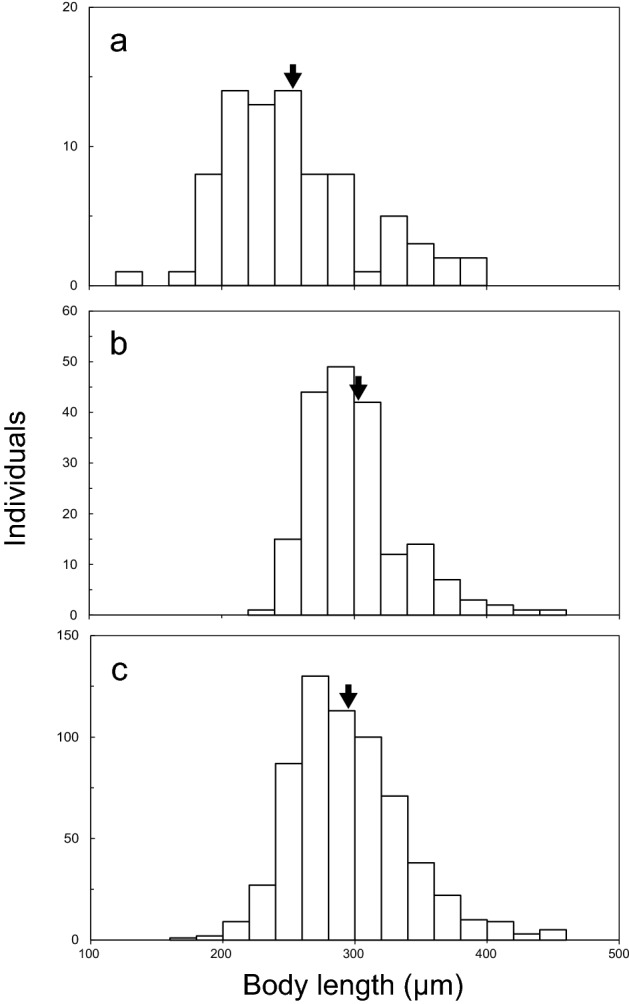
Body size (expressed here as body length) of tardigrades living in green snow (20 µm size increments). (**a**) Collected in April, 2018, (**b**) collected in May, 2018, (**c**) collected in May, 2019. Mean values are shown by the arrows.

The different colors of surface snow on Mt. Gassan were sampled (Figs. 1, 2, 3). Only two groups of microinvertebrates—Tardigrada and Rotifera (Fig. 4 and see supplementary Fig. S1)—were found. Springtails (Collembola) and stoneflies were seen on the surface snow during sampling but they were absent in the collected samples. No other invertebrates (e.g. nematodes) were detected, but abundant ciliates were observed in many samples. Tardigrades were represented by two morpho-species belonging to genus *Hypsibius* with smooth or reticular cuticle (Fig. 4a and supplementary Fig. S2). Both morpho-species had a buccal tube typical for herbivorous species. Rotifers have not been identified to species level; all specimens represented the genus *Philodina* (Fig. 4b). Tardigrades found on the surface snow fell into different size classes, including juveniles (Fig. 5). Exuvia of tardigrades with eggs were found, and contained 4–5 eggs (see supplementary Fig. S3a). Rotifers also covered a range of different sizes (not measured due to their shrinking) along with exuvia containing eggs (see supplementary Fig. S3b).

In moss samples (collected from the bark of trees which are potential habitats for the tardigrades in this forest), tardigrades representing the genera *Hypsibius*, *Macrobiotus* and *Paramacrobiotus* along with heterotardigrades were found. However, based on morphology alone, *Hypsibius* sp. in mosses and on surface snow could not be differentiated. Specimens of *Macrobiotus*, *Paramacrobiotus* and heterotardigrades have not been found in snow during the two seasons.

Body length of tardigrades living in green snow varied in between sampling campaigns (Fig. 5). Their body lengths spanned 129–386 μm (mean ± SD: 253 ± 6 μm) in April 2018 and 226–452 μm (mean ± SD: 302 ± 3 μm) in May 2018, and there were significant differences between April and May (Welch’s t-test, t (113) = 7.54, *P* < 0.001). In May 2019, the range of body length was 178–457 μm (mean ± SD: 298 ± 13 μm), comparable to May 2018, and their mean biomass was $1.1 \pm 1.1 \times 10^{3} \;{\upmu {\rm g}}\;{\text{L}}^{ - 1}$.

### Snow algae and chlorophyll a concentration

**Figure 6:**
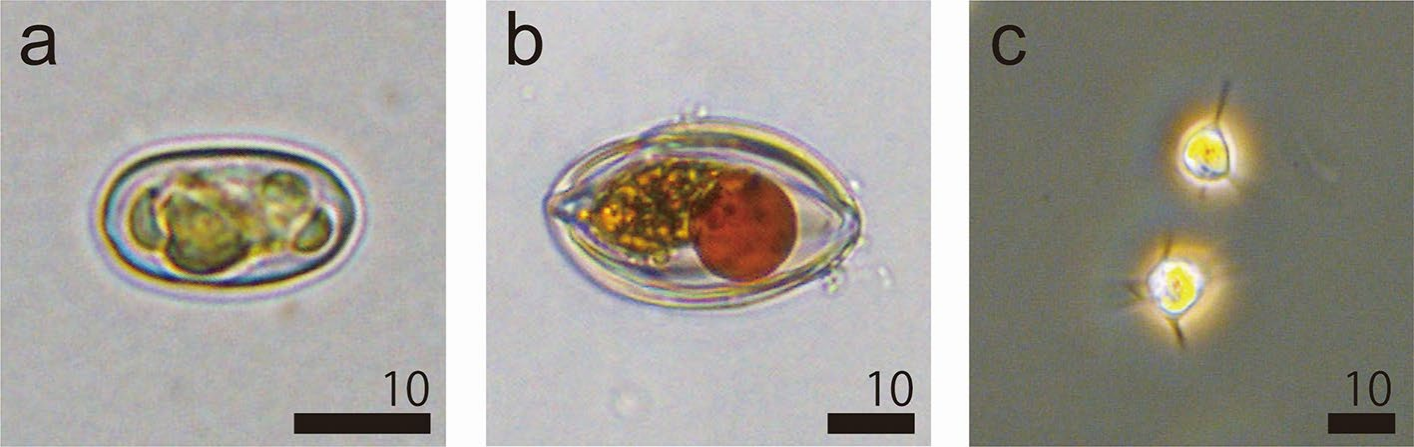
Most abundant snow algae collected in May, 2018. (**a**) Green snow cells (LM: *Chloromonas* sp.), (**b**) orange snow cells (LM: dormant state of *Chloromonas* sp.). (**c**) golden-brown snow cells (PCM: *Ochromonas* sp.). All scale bars in micrometers.

Microscopic observations revealed that each type of colored snow was dominated by morphologically distinct snow algae (Fig. 6). In green snow, oval shaped cells with green chloroplasts were dominating (Fig. 6a). The length of this algae type spanned 13.0–20.1 µm (mean ± SD: 17.2 ± 0.1 μm) and width ranged 7.2–12.7 µm (mean ± SD: 9.2 ± 0.1 μm). In orange snow, oval shaped cells with ribbed cell wall (likely resting stages), green/orange chloroplasts and orange secondary carotenoids were dominating (Fig. 6b). The length of this algae was 36.8–63.4 µm (mean ± SD: 48.2 ± 0.4 μm) and width was 19.9–33.3 µm (mean ± SD: 28.2 ± 1.5 μm). In golden-brown snow, triangular cells with yellowish chloroplasts were dominating (Fig. 6c). The length of this algae was 7.5–10.7 µm (mean ± SD: 9.1 ± 0.9 μm).

**Figure 7:**
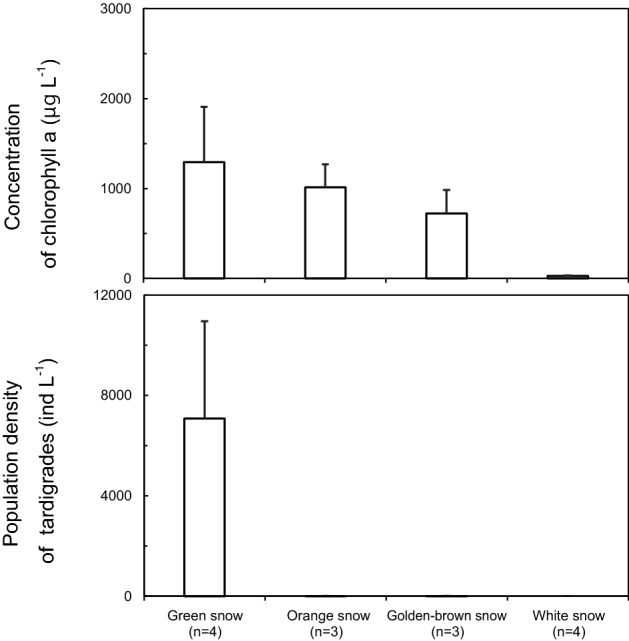
Population density of tardigrades and concentration of chlorophyll *a* per meltwater volume in each color type of snow collected in May, 2018. n: number of snow samples, error bar—standard error.

**Figure 8:**
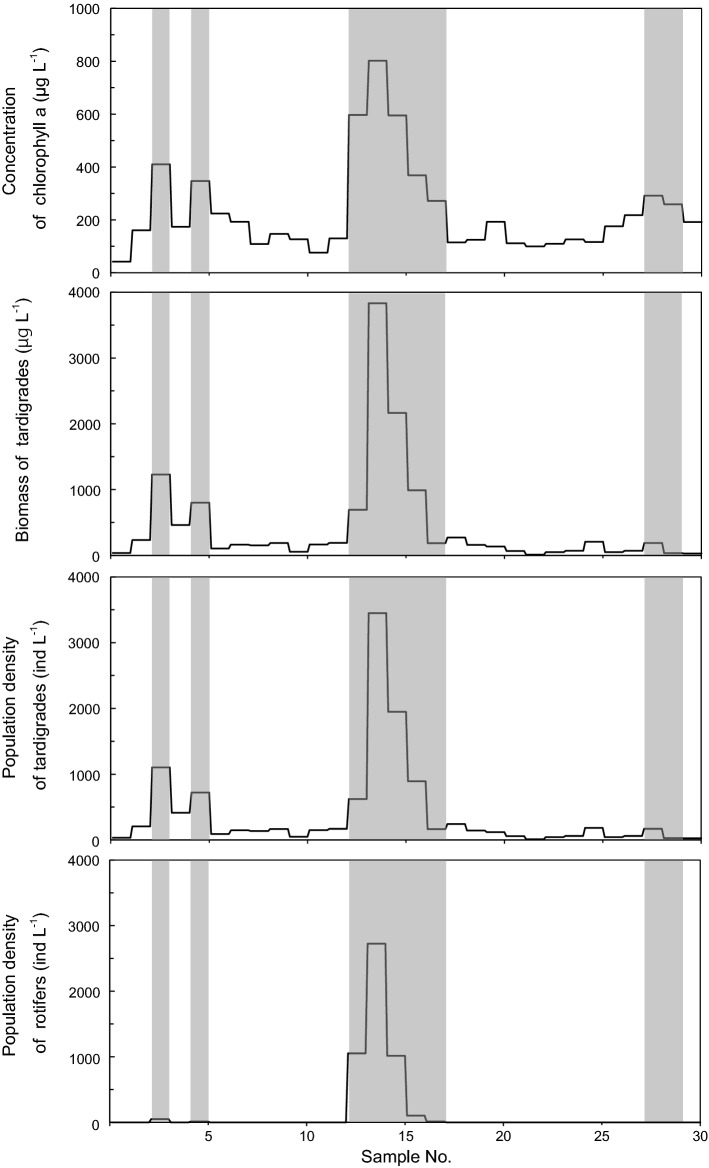
Concentration of chlorophyll *a*, calculated dry biomass of tardigrades and population density of tardigrades and rotifers per meltwater volume collected along a lateral line on snow surface in May 2019. Green snow is shown as gray zones.

Chlorophyll *a* concentration, as an indicator of abundance of photoautotrophs, largely varied across the snow surface. Concentration of chlorophyll *a* was always higher in colored snow (Figs. 7, 8, Table 1). In samples from April and May 2018, chlorophyll *a* concentration was $2.9 \pm 1.3 \times 10^{2} \;{\upmu {\rm g}}\;{\text{L}}^{ - 1} \left( {{\text{mean}} \pm {\text{SD}}} \right)$ and $1.3 \pm 0.6 \times 10^{{3{ }}} \,{\upmu {\rm g}}\;{\text{L}}^{ - 1}$ in green snow (April and May respectively), $1.0 \pm 0.4 \times 10^{3} \;{\upmu {\rm g}}\;{\text{L}}^{ - 1}$ in orange snow (May), $7.2 \pm 4.5 \times 10^{2} \;{\upmu {\rm g}}\;{\text{L}}^{ - 1}$ in golden-brown snow (May) and $28 \pm 5\;{\upmu {\rm g}}\;{\text{L}}^{ - 1}$ in white snow (May). In samples from May 2019, chlorophyll *a* concentration were $4.4 \pm 1.8 \times 10^{2} \;{\upmu {\rm g}}\;{\text{L}}^{ - 1}$ in green snow and $1.4 \pm 0.5 \times 10^{2} \;{\upmu {\rm g}}\;{\text{L}}^{ - 1}$ in white snow. There were no significant differences in the chlorophyll *a* concentration between green snow and other colored snow, but there were significant differences between white snow and colored snow (supplementary Table S1).

**Table 1:** Number of snow samples, population density of microinvertebrates and concentration of chlorophyll *a*.

Year	Month		Snow patches			
			White	Green	Orange	Golden-brown
2018	April	n	4	4	N/A	N/A
		Inv	0, –	7.3 × 10^3^, –	N/A	N/A
		Chl-a	0	2.9 × 10^2^	N/A	N/A
	May	n	4	4	3	3
		Inv	0, –	7.1 × 10^3^, –	7, #	7, #
		Chl-a	28	1.3 × 10^3^	1.0 × 10^3^	1.0 × 10^3^
2019	May	n	21	9	N/A	N/A
		Inv	1.2 × 10^2^, 0	1.0 × 10^3^, 5.5 × 10^2^	N/A	N/A
		Chl-a	1.4 × 10^2^	4.4 × 10^2^	N/A	N/A

Inv.: population density of microinvertebrates (tardigrades or rotifers, ${\text{ind}}\;{\text{L}}^{ - 1}$), Chl-a: concentration of chlorophyll *a* (${\upmu {\rm g}}\;{\text{L}}^{ - 1}$), N/A: colored snow was absent, # : rotifers were present but not counted.

### Relationship between microinvertebrates and snow algae

Tardigrades and rotifers mainly occurred in green snow and their population densities were different between snow colors. In April and May 2018, population densities of tardigrades were $7.3 \pm 2.9 \times 10^{3} \;{\text{ind}}\;{\text{L}}^{ - 1}$ (mean ± SD), $7.1 \pm 3.9 \times 10^{3} \;{\text{ind}}\;{\text{L}}^{ - 1}$ in green snow (April and May respectively), $7 \pm 2\; {\text{ind}}\;{\text{L}}^{ - 1}$ in orange snow (May), $7 \pm 2\;{\text{ind}}\;{\text{L}}^{ - 1}$ in golden-brown snow (May) and 0 ${\text{ind}}\;{\text{L}}^{ - 1}$ in white snow (May) (Fig. 7 and Table 1). In May 2019, population densities of tardigrades and rotifers were $1.0 \pm 0.4 \times 10^{3} \;{\text{ind}}\;{\text{L}}^{ - 1}$ and $5.5 \pm 0.6 \times 10^{2} \;{\text{ind}}\,{\text{L}}^{ - 1}$ in green snow, $1.2 \pm 0.2 \times 10^{2} \;{\text{ind}}\;{\text{L}}^{ - 1}$ and 0 ${\text{ind}}\;{\text{L}}^{ - 1}$ in white snow, respectively (Fig. 8 and Table 1). In green snow, population densities of tardigrades, rotifers and concentration of chlorophyll *a* were significantly correlated (Pearson's correlation coefficient: tardigrades and rotifers, r = 0.91, *P* < 0.01; tardigrades and chlorophyll *a*, r = 0.87, *P* < 0.01; rotifers and chlorophyll *a*, r = 0.84, *P* < 0.01). Tardigrades also had remains of algal cells in their intestines and their intestines were colored green (Fig. 4a and supplementary Fig. S1).

## Discussion

Over two studied seasons in beech forests in Japan, tardigrades and rotifers were concentrated on seasonal melting snow patches, particularly in green snow. The observations suggest that snow algae blooms are beneficial for particular species of tardigrades and rotifers. On the snow surface, two tardigrade taxa belonging to genus *Hypsibius* were determined based on morphology and rotifers represented *Philodina* genus (Fig. 4). The high densities of microinvertebrates, their prevalence over two seasons and a wide range of size classes found (Figs. 5, 7, 8) suggest that tardigrades and rotifers thrive and are active on the seasonal snow patches and have adapted to snow surface environments. Tardigrades of the genus *Macrobiotus* (known to feed on algae in laboratory cultures^37^), and *Paramacrobiotus* (predators in natural environment^38^) were found in tree mosses present at the study site, but they were absent in surface snow. Their presence on seasonal snow patches is likely limited by physiological or dispersal constrains. A temperature regime might be a major factor as it seems to shape the tardigrade communities on glaciers^25^. Constant low temperature on the snow surface might inhibit growth and reproduction of some tardigrade species, but benefit the others which need low temperatures for reproduction. Factors which may explain the benefits of living on the snow include: (a) microinvertebrates have stable and abundant food source (snow algae), (b) predators for microinvertebrates such as spiders and mites are rare.

The source of *Hypsibius* species on surface snow remains unclear. Most probably, microinvertebrates on surface snow are airborne, passively transported by wind from distant locations, or, more probably, gravitationally from tree canopies. Hypothetically, if they would be passively transported to surface snow from surrounding mosses from the tree trunks, *Macrobiotus*, *Paramacrobiotus* or heterotardigrades should be found on surface snow not only two *Hypsibius* ssp. Previous work conducted in Akashibo snow patches in Japan, reported tardigrades and other microinvertebrates (e.g. Nematoda, Oligochaeta) in snow layer under the snow patches (inside the snowpack), which probably migrated from soil. However, these microinvertebrates did not seem to be active in snow e.g. they did not grow or reproduced^39^. Taking this into account, a simple life cycle of microinvertebrates can be hypothesized (I): With appearing snow, microinvertebrates are transported to snow surface by wind from nearby habitats not covered by snow, or gravitationally from canopies; only species that require or are able to be active, feed and reproduce on snow will survive (April), (II): microinvertebrates grow and reproduce thanks to feeding on green snow algae (May), (III): after complete snow melt, they are deposited on mosses (no snow season).

Although there were no differences in concentrations of chlorophyll *a* between all the types of colored snow (Fig. 7), it seems that green snow provides the most favorable conditions for growth and reproduction of tardigrades and rotifers in the study area. In green snow, exuvia of tardigrades and rotifers were found (supplementary Fig. S3) and mean body length of tardigrades in May 2018 was bigger than in April 2018 (Fig. 5) which suggests ongoing growth. Simultaneously, concentration of chlorophyll *a* in snow also increased with time, providing a continuous source of food increasing numbers and sizes of tardigrades.

Population density of microinvertebrates was related to green snow algae abundance, but not other algae, which may result from the feeding preferences. Tardigrade taxa found in green snow are typical algae feeders^37,40^ and indeed the intestines of specimens in this study were green (Fig. 4a and supplementary Fig. S1). The diet of rotifers could not be concluded from microscopic observations, but it is possible that rotifers feed on algal cells or the bacteria or yeasts associated with algal blooms. The algae genera well known to color the snow red (*Chloromonas* spp. or *Sanguina* spp.) were previously reported in Canada^5^. The authors presented tardigrades, rotifers, mites and springtails in the studied red snow, and showed that both tardigrades and rotifers had the same red intestine content. No animal density were available for comparison. On Mt. Gassan, snow algae which are present in green and orange snow, are a mix of several species of genus *Chloromonas*, whereas c.f. *Ochromonas* species are common in golden-brown snow^10,11,41,42^. Even though green and orange snow usually contains the same *Chloromonas* species, the difference in their color is mostly due to the difference in life cycle. In green snow, algal cells are mostly motile vegetative cells, which have chloroplasts without any secondary pigment. They can asexually reproduce in the melting surface snow and color the snow green. In contrast, algal cysts have ribbed and thick cell walls, and contain orange or red colored secondary carotenoids (e.g. astaxanthin). Such secondary pigments are usually produced when snow algae are stimulated by light, for minimizing the amount of light^42,43^. Herbivorous tardigrades are thought to eat algae by sucking^37^, so thick cell walls of orange snow algae might prevent tardigrades from eating them. Moreover, tardigrades in laboratory cultures fed on *Chlorella vulgaris* and *Chlorella* sp.^44,45^ which have similar round shape and size to green snow algae (2–10 µm for *C. vulgaris*^46^). In case of golden-brown snow algae, their cell walls and size do not seem to interfere with the tardigrade’s feeding mode, but instead these algae may be toxic, similarly to reports from coastal waters^47^. Therefore, it seems possible that tardigrades get something beneficial from contents of green snow algae (ex. pigments, carbohydrates, amino acids). However, the underlying mechanisms require further testing in the future.

Snow patches serve as a promising arena for studies on biodiversity and ecology of these ubiquitous microinvertebrates. Further studies on the consumption rates of snow algae by consumers may bring a new data on how invertebrates suppress algal blooms and how these algae respond to abundant invertebrates. Understanding the relationship between snow and biosphere including microinvertebrates is an important factor in predictions of future changes and biogeocycles in snow ecosystems.

## Material and methods

### Study site

The field study was conducted at Yumihiradaira park (39° 30′ N, 140° 00′ E: altitude 770 m above sea level (a.s.l.)) on Mt. Gassan, Yamagata prefecture in Japan (Fig. 1). The peak of Mt. Gassan has an altitude of 1984 m a.s.l. and the main mountainous ridge extends from the north to the south along the west coast of the northern part of the Honshu Island. Due to the monsoon, there is a lot of snow in this area^48^. Vegetation at the study site is dominated by mountain broad-leaved deciduous trees (including beech trees shown in Fig. 1) up to the elevation of 1500 m a.s.l. and above this elevation, subalpine bamboo, ash and maple dominate up to the top of the mountains^48^.

The study site is covered with snow from mid-November until early June. The snow depth usually reaches approximately 4 m of maximum depth in February. According to the meteorological station near the study site (750 m a.s.l.), which is operated by Snow and Ice Research Center, National Institute of Disaster Research in Japan, the daily mean air temperature ranges from approx. – 8 °C to 4 °C in January and February (reached approx. 17 °C during sampling).

### Sampling

For analysis of microinvertebrates and snow algae, surface snow and moss samples were collected on April 23rd, May 14th 2018 and May 18-20th 2019 at the same site surrounded by the beech trees (Fig. 1). Snow depths in the study site were approx. 320 cm, 163 cm, and 90 cm, respectively. A total of 52 surface snow samples (the distances between snow samples were within a radius of 200 m) and 8 small (few grams of dry weight) moss samples attached to beech tree trunks were collected. The dimensions of the collected snow surface was 10 × 10 × 2 cm (length × width × depth). During the study periods, there were various colors of snow surfaces, and the colored patches had sizes of about 10 cm to 30 cm in diameter. We classified the snow surface in 4 different coloration types, which were visually identified as white, green, orange, and golden-brown snow as described before^41^. The different snow colors were most likely shaped by abundance and species composition of snow algae. All four different colors of snow were collected in the season of 2018 (white snow was selected randomly, Fig. 2), white and green snow was collected along the lateral line set in 2019 (Fig. 3). In order to compare microinvertebrate taxa with those in the closest snow free habitats, moss samples were collected in 2018 (April = 4 samples, May = 4 samples). All snow samples were collected by using sterile stainless-steel scoop, and all moss samples were collected by using a small shovel. Immediately after sampling, material was frozen and kept in 50 ml polypropylene bottles (AS ONE Corporation, Osaka, Japan) or Whirl–Pak bags (Nasco, Fort Atkinson, WI, USA). All samples were transported to the laboratory at Chiba University, Japan and stored in a freezer (− 20 °C) until further processing.

### Microscopic observation of microinvertebrates

Surface snow and moss samples were melted gradually at refrigerator (5 °C) in order to avoid potential thermal shock for psychrophilic invertebrates. After melting, liquid water was moved directly to a petri dish and examined using a MZ125 stereo microscope (Leica Microsystems, Wetzlar, Germany) with FLEXACAM C1 digital camera (Leica Microsystems, Wetzlar, Germany). Moss samples were put in a petri dish, MiliQ water was added and left for 2–3 days to wait for animals’ recovery. After 2–3 days, mosses were removed by shaking, using tweezers. Then, the big particles have been mechanically removed and water with fine sediment was analyzed. Invertebrates were counted and put into 6 ml glass tubes (AS ONE Corporation, Osaka, Japan) with 70% ethanol for preservation. Some individuals photographed using a BX51 phase contrast microscope (Olympus, Tokyo, Japan) with DP21 digital camera (Olympus, Tokyo, Japan). Population density of tardigrades and rotifers in surface snow was calculated based on tardigrades or rotifers counts per volume of melted water.

Tardigrades and rotifers were mounted on microscope slides in a small drop of Hoyer’s medium^49^ or in drop of water then examined under the following light microscopes BX51 and Olympus BX53 with phase-contrast (PCM) and another with differential interference contrast (DIC). Pictures were taken using a DP21 digital camera, cellSens Entry 1.12 software (Olympus, Tokyo, Japan) or Quick PHOTO CAMERA 3.0 software (Promicra, Prague, Czech Republic). For species identification the keys and species descriptions were used for each tardigrade^50,51^ and rotifer^52^. For calculation of biomass of tardigrades, their body length and width was measured on the pictures from Olympus BX53 and measured in Image J 1.52 software^53^ and Quick PHOTO Camera 3.0 software (Promicra, Prague, Czech Republic). The animals not suitably orientated for morphometry (shrunken or bent) were removed from analysis. Body length was measured without length of legs IV and width of the body was taken between legs II and III. Biomass (dry weight, W) of each specimens was calculated based on formula^54^: if body length (L) and width (D) were 4:1; ${\text{W}} = {\text{L}}^{3} \times 0.051 \times 10^{ - 6}$, 5:1; ${\text{W}} = {\text{L}}^{3} \times 0.033 \times 10^{ - 6}$. Body lengths of each morpho-species were overlapping and their averages were not significantly different (U = 158, *p* > 0.05), thus biomass for tardigrades was calculated for all the types together.

### Microscopy of snow algae and measurements of chlorophyll a concentration

Melted snow was used for microscopic observation of snow algae and determination of the chlorophyll *a* concentration serving as a proxy for abundance of algae^55^. Around 20–50 µm of melted water with algae was photographed using a BX51 phase contrast microscope with DP21 digital camera. Length and width of snow algae was measured in Image J 1.52 software^53^. For measurements of the chlorophyll *a* concentration, Welschmeyer fluorescent method was used; melted water (approx. 90–150 mL per sample) was filtered through a glass micro filter (Whatman glass microfiber filters gf/f 25 mm, Cytiva, Tokyo, Japan) into 8 ml sterilized tube (60.542.024, Sarstedt, Nümbrecht, Germany) with 6 ml of N,N-dimethylformamide (DMF) to extract chlorophyll *a*. After 2–3 days of incubation in the fridge to allow pigment extraction, fluorescence intensity was measured by using a *Trilogy Laboratory Fluorometer* (Turner, CA, USA). Chlorophyll *a* concentrations were calculated from fluorescence intensity based on molecular extinction coefficient^56^ (88.74 L^−1^ g^−1^ cm^−1^), and a calibration curve created by measuring fluorescence intensity and absorbance (664 nm) of *chlorophyll a from spinach* (C5753-1MG, Sigma-Aldrich Japan, Tokyo, Japan) with a fluorometer and a spectrophotometer (UV-mini 1240, Shimadzu, Kyoto, Japan).

### Statistical analysis

To test differences between invertebrate biomass and densities, and chlorophyll *a* in white and colored snow and seasonal changes of tardigrade body size, two statistical tests were used. The nonparametric Mann–Whitney U test for testing differences of population densities of tardigrades and rotifers or concentration of chlorophyll *a* between white and colored snow, and the Welch’s t-test for testing differences in seasonal changes of body length of tardigrades. All statistical analysis were calculated using R software^57^.

## Supplementary Information

Supplementary Information
