# Supplementary material for: Snow algae blooms are beneficial for microinvertebrates assemblages (Tardigrada and Rotifera) on seasonal snow patches in Japan
Source: Sci Rep. 2021 Mar 16;11:5973. doi: 10.1038/s41598-021-85462-5 (PMC7971028; doi:10.1038/s41598-021-85462-5)
Supplement: Supplementary file 1 — Supplementary Information [file 41598_2021_85462_MOESM1_ESM.pdf]

Title of the manuscript: Snow algae blooms are beneficial for microinvertebrates assemblages (Tardigrada and Rotifera) on seasonal snow patches in Japan

Authors: Masato Ono[1*], Nozomu Takeuchi[2], Krzysztof Zawierucha[3]
[1] Graduate School of Science and Engineering, Chiba University, Chiba, Japan
[2] Department of Earth Science, Graduate School of Science, Chiba University, Chiba, Japan
[3] Department of Animal Taxonomy and Ecology, Adam Mickiewicz University, Poznań, Poland

This file includes three supplementary figures. Legends are below and all figures are in next page.

Supplementary Figure S1. Microinvertebrates and snow algae in green snow (SM, black background). Scale bar is in micrometer.

Supplementary Figure S2. Cuticle of *Hypsibius* **sp.** (PCM). (a) smooth cuticle, (b) reticular cuticle. All scale bars in micrometers.

Supplementary Figure S3. Exuvia of microinvertebrates with egg. (a) *Hypsibius* **sp.** (LM), (b) *Philodina* **sp.** (PCM). All scale bars in micrometers.

Supplementary Table S1. U Mann-Whitney values and significance level for chlorophyll *a* among concentration of white and colored snow. Significant values are marked in bold. N/A: could not see colored snow (orange or golden-brown).

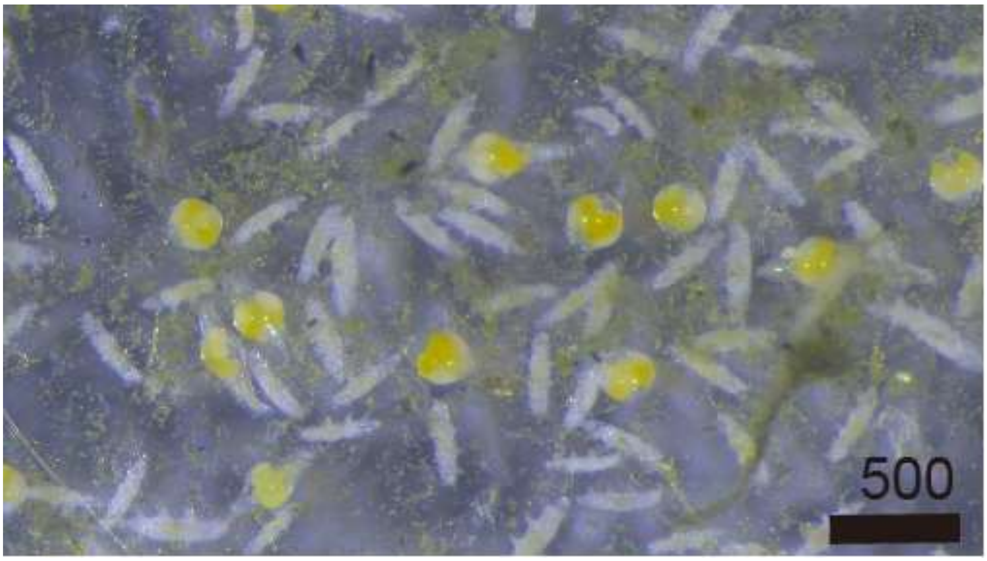

Supplementary Figure S1. Microinvertebrates and snow algae in green snow (SM, black background). Scale bar is in micrometer.

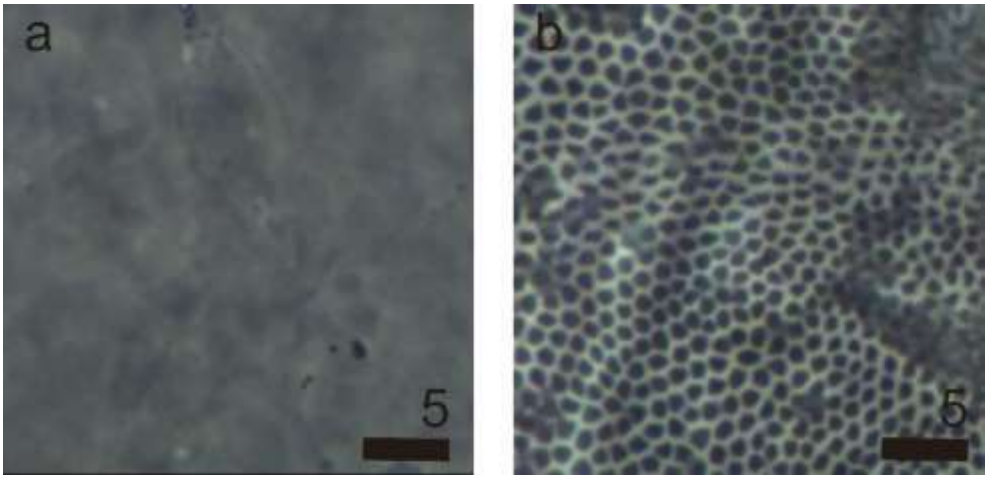

Supplementary Figure S2. Cuticle of *Hypsibius* **sp.** (PCM). (a) cuticle skin, (b) reticular skin. All scale bars in micrometers.

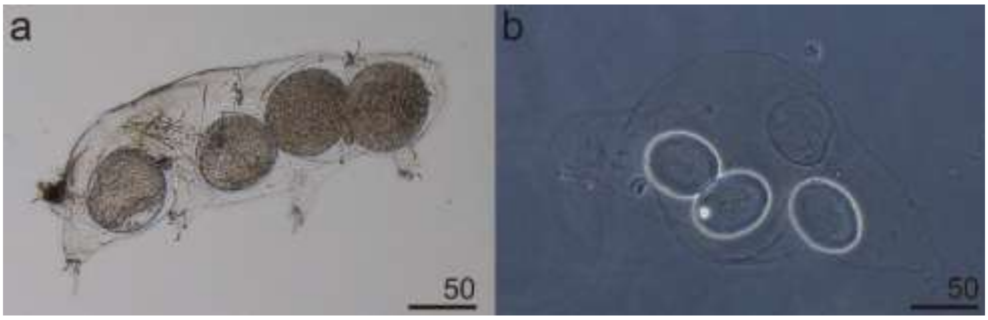

Supplementary Figure S3. Exuvia of microinvertebrates with egg. (a) *Hypsibius* **sp.** (LM), (b) *Philodina* **sp.** (PCM). All scale bars in micrometers.

Supplementary Table S1. U Mann-Whitney values and significance level for chlorophyll *a* among concentration of white and colored snow. Significant values are marked in bold. N/A: could not see colored snow (orange or golden-brown).

Year	Month	Snow patches				
		White vs Green	White vs Orange	White vs Golden-brown	Green vs Orange	Green vs Golden-brown
2018	May	**U = 0, P < 0.05**	**U = 0, P < 0.05**	**U = 0, P < 0.05**	U = 6, P > 0.05	U = 5, P > 0.05
2019	May	**U = 0, P < 0.001**	N/A	N/A	N/A	N/A
